# Case of Invagination of the Colon in the Rectum

**Published:** 1804-02-01

**Authors:** G. Roux


CASE OF INVAGINATION OF THE COLON IN THE RECTUM; *read at the Medical Society of Paris, by G. Roux, M.D. Communicated by our Correspondent at Paris.*

A. B. a labourer, forty-eight years of age, was seized in the spring with diarrhœa, accompanied by derangements of the stomach. The day of its occurrence he had committed some excesses as well in the use of drink as of exercise; the patient complained of cholic pains, which were the consequence of this excess; in short, the complaint increased in intensity and violence, so as to oblige him to go twenty or twenty-five times a day to stool. During one of those efforts a tumour appeared of the size of an egg, exteriorly, at the anus, accompanied by such violent pain as to render it impossible for the patient to stand erect; in this state he was got to bed, and in less than two hours the tumour had increased very considerably. A woman, who happened to be present, made many useless efforts to reduce the tumour, after which the patient called for medical assistance.

The person who was consulted did not endeavour to reduce the swelling, but had recourse to general bleeding and the use of barley water; the bleeding was repeated the same evening. The third day of the existence of the complaint, M. Roux, and M. Lauernet, surgeons, were called to see the patient, who found the fever considerable and the belly painful to the touch; pains were also felt, which

I 2 extended

---

*end of all amicable discussion*, being long since assured that the real interests of truth have nothing to apprehend from the closest severity of investigation, nor the utmost censure human judgment can pronounce."

extended from the epigastric region down to the extremity of the tumour, which had now a frightful appearance; it was from nine to ten inches in length, and six or seven broad, of a chesnut colour, hard, and extremely painful, and furrows of a deep brown colour were observable on its surface. The size and hardness of the tumour precluded the possibility of its reduction, and the gentlemen in attendance confined themselves to the use of such means as were best suited to calm and allay irritation. The use of the white decoction of Sydenham was recommended as drink, and baths procured some relaxation from pain, which, as well as the tension, had much diminished; the colour of the swelling, however, was become much darker.

On the fourth day a hiccup commenced, which increased considerably in the course of the day; the fever and pains in the intestines continued, and the appearances, as already described, remained the same, as well as the impossibility to effect a reduction. The same treatment was continued.

On the fifth day the hiccup and pyrexia had increased; and in the evening nausea and vomiting came on. The tumour was flaccid in some places, but the circumference of the anus continued hard; and the exterior membrane of the tumour seemed disposed to exfoliate. New and fruitless efforts were made to reduce it. From this moment all hopes of future success were given up; "we even doubted its utility or advantage, for we had reason to presume the existence of a strangulation above sufficiently great to have caused gangrene to be the principal source of the accidents which now began to succeed each other with rapidity, and which bore a striking analogy with the appearances of strangulated hernia. We determined to make use of antispasmodics united to antiseptics." A Note of the Author observes, that "the very critical situation in which he, as well as the other persons in attendance, were placed, required every precaution. It was at this period that we examined the question, Whether an enlargement of the anus, with a view to favour reduction, was advisable in the present state of things? We inclined to the negative so much the more strongly, as we were really ignorant of the parts engaged, or of the seat of the strangulation."

On the sixth day, all the symptoms had increased, accompanied by vomiting of stercoral matter; the pulse was weak, small, but regular; the tumour was flaccid, and there was a sensible diminution of volume; there issued from it a very strong putrid exhalation, accompanied by an ichorous

-ous oozing from its surface, and about the verge of the anus.

On the seventh day, the fever was stronger in the morning, the pains in the belly more sharp, respiration more laborious, as well as the vomiting and hiccup; the tumour was much diminished, but the tension about the sphincter continued. The medical attendants having communicated the approaching death of the patient to his friends, they gave him up to the care of an old woman, who, by repeated efforts, had thrust the tumour within the rectum. In the evening of this day the patient died.

Persuaded of the existence of a volvulus, M. Roux, &c. obtained, after long solicitation, permission to examine the body; and the following were the appearances: "The external tumour had almost entirely disappeared; on opening the abdomen much gas issued, and there was found in the cavity about a quart of a serous brownish-coloured fluid. The volume of intestines was greater than natural, and they were covered by a new membrane, which connected them with the peritoneum on all sides. The intestinum ileum, two-thirds of the superior portion of the colon and the cæcum were inflamed, particularly the two first. Pursuing our inquiries toward the rectum, we perceived, at the superior part of this intestine, an inflammatory ring, about seven or eight inches distant from the sphincter, and compressing very strongly the inferior part of the colon. While we were examining these appearances, we remarked the laceration of the false membrane that united the two surfaces of the intestines, evidently invaginated. The rectum, when opened above the ring in question, shewed the colon invaginated in the rectum nearly thirteen inches. The two superior inches of this portion of the intestine bore evident marks of an alteration of structure. The diameter of the intestine was much contracted at this place, and its parietes were very much thickened; the three inches below this, and which formed nearly a third of the invagination, were free, and had scarcely any appearance of disease. The two inferior thirds of the invagination offered all the appearances of the intestinal tumour already remarked to have formed the external swelling: And, lastly, the two extremities of the invagination adhered strongly to the corresponding points of the rectum. The mucous membrane of the rectum was much tumefied, and altered from its natural state."

"These appearances (continues the Author) demonstrate to evidence the existence of intus-susceptio, a disease noticed by the Father of Medicine, and on which Morgagni has much insisted." We find in Albinus, Fabricius, Le Cat, and Sabatier, very exact notions on the nature of this disease, particularly the latter, who, in a Paper inserted in the Memoires de l'Acad. tom. v. speaking of the supposed falling down of the rectum, says, "These facts prove that the disease called the descent of the anus or rectum, instead of being produced by the inferior part of the intestine, or by the prolongation of its internal coat tumefied, as has been hitherto supposed, has been frequently the effect of a volvulus or invagination of the intestinal canal, beginning at a greater or less distance from the anus, and which, after having forced through this opening, presents itself externally.

M. Roux asks, if art offers any resource in this case? He thinks it would be useless, if not injurious, since the strangulation does not always exist at the anus, and that we know not the place of its real existence. Should excision be proposed? Would not an operation, which comprehends parts of such great importance be inadmissable? Should an incision the length of the tumour be made? What would be its object? or what could be gained by it? And, lastly, he asks, Should gastrotomy be resorted to? This resource, proposed by some authors, and rejected by others, is but momentary; for considering the danger of the disease, and comparing its consequences with its disadvantages, we are obliged to confess with Hiver, that it should be banished from Surgery. Would the reduction of the tumour remedy the fatal consequences of invagination? This is probable; but it should be effected immediately, half an hour or an hour might give rise to changes which would render the operation abortive. Let us suppose that it was reduced under proper circumstances, would it not be useful, observes M. Roux, after reducing the intestine, to pass the index finger, with a view of disengaging all the invaginated intestine; after which emollient clysters, &c. should be employed. In this particular, the Author's situation, as well as of other medical men in attendance, precluded the possibility of every effort, and his motive in making it public, is to throw some light on the subject, and to call the attention of practitioners to similar occurrences.

The Revisors of this article, Messrs. Sedillott and Giraud, give the following Note on this Case. We think, say they, that in the cases where the reduction is impossible, that Gastrotomy, properly practised, becomes necessary.

It would have been useful, they add, if the author had dwelt more on the state of the intestines, and that he had informed us whether their structure was not torn, in order to give passage to the intestine forming the tumour, so as to be contained only by the mucous membrane, as we observed in the case of a child of seven years old, in which all the small intestines protruded at the anus.

*Paris, Nov.* 17, 1803.

*** We recommend a paper on this subject by M. Fages, inserted in the 7th vol. of our Journal; in which will be found the opinions on the advantages and disadvantages of Gastrotomy. Ed.

